# Psychogenic Non-epileptic Seizure in a Laboring Female: A Case Report

**DOI:** 10.7759/cureus.67218

**Published:** 2024-08-19

**Authors:** Alexandria Sobczak, Randy Felber, Alyson Skelly, Hemangi Patel, Samuel Falzone

**Affiliations:** 1 Dr. Kiran C. Patel College of Osteopathic Medicine, Nova Southeastern University, Fort Lauderdale, USA; 2 Obstetrics and Gynecology, Wellington Regional Medical Center, Wellington, USA

**Keywords:** psychogenic non-epileptic seizures, case report, delivery and labor, seizure, pregnancy, psychogenic non-epilipetic seizures

## Abstract

Psychogenic non-epileptic seizures (PNES) are seizure-like activities characterized by motor and sensory impairments that are mild and mimic other medical conditions. They are commonly associated with psychiatric conditions and are typically a diagnosis of exclusion. These episodes are generally uncommon and rarely seen in pregnancy or labor. The treatment consists of managing the underlying cause as well as cognitive behavioral therapy. They may mimic absence seizures, which are diagnosed when there are episodes of staring present. This report presents the case of a 26-year-old laboring female who experienced multiple psychogenic seizures. The purpose of this case report is to illustrate how psychogenic non-epileptic seizures (PNES) can imitate epileptic or absence seizures and, thus, should be a diagnosis considered in females in labor.

## Introduction

While seizures are known to be a common complication in pregnancy, especially in previous epileptic patients, little is known about the role psychogenic seizures play in pregnancy and labor [1]. According to the Diagnostic and Statistical Manual of Mental Disorders, Fifth Edition (DSM-5), psychogenic non-epileptic seizures (PNES) is classified as a somatic symptom illness and is specifically a conversion disorder within the psychiatric field. PNES is characterized by subtle motor or sensory impairments that go unnoticed or by symptoms that mimic other medical conditions and remain undiagnosed as neurological conditions. These symptoms result in clinically significant distress or functional status impairment [2].

Psychogenic seizures are estimated to be present in 2 to 33 per 100,000 individuals and are somatic displays of an underlying psychological issue [3]. These seizures tend to present more commonly in female patients in their 20s and 30s [4] and in patients with other psychiatric conditions such as PTSD, bipolar, depression, and anxiety [5]. Physical and psychological trauma, as well as abuse and cognitive impairment, are other known risk factors [6].

Clinically, during a psychogenic seizure, different subtypes of behavior may be noted such as convulsions, collapse, and lack of responsiveness to stimuli including pain [3]. This can be similar to the presentation of epileptic seizures, which are caused by abnormal electrical activity in the brain [6]. In addition, psychogenic seizures can mimic absence seizures, which are a specific type of epileptic seizure characterized by brief episodes of staring and unresponsiveness [7]. Although difficult to distinguish at the bedside, PNES seizures will lack the abnormal EEG findings observed during epileptic seizures [6]. Any signs of unresponsiveness in a female in labor are concerning, given the risk of compromised oxygenation and blood flow to the fetus.

To our knowledge, the prevalence of PNES in pregnancy, labor, and during the postpartum period is not well-documented in the literature. This case report presents a rare case of PNES in a female in labor.

## Case presentation

A 26-year-old Gravida 2 Para 1 female presented to labor and delivery at 40 weeks and 5 days gestation for induction of labor due to oligohydramnios. The patient’s past medical history included a history of ovarian cysts, psychogenic hyperventilation, and obesity. On admission, the patient’s vitals included a temperature of 97.9F, heart rate (HR) of 82 beats per minute (bpm), respiratory rate of 16 breaths per minute, oxygen saturation (SpO2) of 98%, and blood pressure (BP) of 108/56 mmHg.

Twelve hours after being admitted to labor and delivery for induction, the patient suddenly became unresponsive to verbal and physical stimuli. Due to this acute mental status change, the patient was taken emergently to the operative room for a cesarean section. After the initial anesthesiology evaluation, the decision was made to intubate the patient, as she was prepped for emergent surgery. 

During cesarean section, the patient was found to have placenta accreta, a condition in which the placenta is abnormally attached to the uterine wall. Due to significant intraoperative hemorrhage from the accreta, the patient was transfused with three units of packed red blood cells. After completion of the cesarean section, the patient was transferred while intubated to the critical care unit for further management. The anesthesiologist was concerned that she would not be able to protect her airway while still unresponsive, so she remained intubated.

Her vital signs from the time of cesarean section until the following morning ranged from temperature 97.8-98.7F, HR 79-99 bpm, BP 95-130/53-81 mmHg, mean arterial pressure (MAP) 76-96 mmHg, and SpO2 97-100%.

On postoperative day one, the patient regained consciousness and was extubated. When asked what she recalled from the previous night, the patient reported having severe pain during labor contractions, diffuse body paresthesia, and lightheadedness. On further questioning, she also reported a similar incident of unresponsiveness during the pregnancy of her first child. During her prior episode, she states she had a full workup including labs, echocardiogram (ECHO), and electroencephalogram (EEG) during her symptomatic and asymptomatic phases, and magnetic resonance imaging (MRI) of the brain, which were all within normal limits. However, she cannot attest to any further follow-up or formal diagnoses during the previous episode.

An extensive workup was performed to rule out potential causes of her unresponsiveness. Ultrasound of the carotid arteries showed no evidence of stenosis. Her EEG was within normal limits. Her echocardiogram (ECHO) was normal and depicted no abnormalities. MRI of the brain and cervical spine also showed no significant findings. Prior to delivery, the patient was unresponsive for 15 minutes and remained unresponsive for several hours after surgery. The syncopal episode was unlikely due to the duration of unresponsiveness and the absence of positional changes. Notably, her lack of consciousness was not associated with hypotension, as her blood pressure remained within normal limits, and positional changes did not aid in regaining consciousness.

On postoperative day two, less than 36 hours after her first unresponsive episode, the patient complained of lightheadedness and severe pain before again enduring intermittent episodes of changes in mental state. She became tremulous, and confused but was arousable and able to follow commands. After regaining awareness one hour later, she reported feeling chest pressure, dyspnea, and anxiety prior to the episode. EEGs performed after the first episode and during the second episode were normal. Conditions relating to cardiac and neurological causes were ruled out with extensive workup including EEG, ECHO, MRI brain and cervical spine, computed tomography angiogram (CTA), and transthoracic echocardiogram (TTE) with Doppler imaging. Complete blood cell count, comprehensive metabolic panel, and blood gas analysis were all within normal limits. The neurological exam was unremarkable. Imaging was without any abnormalities. Throughout both episodes of mental status change, her vitals remained stable.

The patient remained in observation for several days and no new episodes of acute mental status change occurred. On postoperative day five, her vitals were stable with an HR of 68 bpm, temperature of 97.9F, respiratory rate of 18 breaths per minute, and BP of 110/68 mmHg. The patient was discharged on levetiracetam 500 mg two times a day orally and told to follow up with a neurologist. The patient was also given follow-up instructions from the obstetrician and gynecologist (OBGYN).

## Discussion

Psychogenic seizures are estimated to be present in 2 to 33 per 100,000 individuals and are somatic displays of an underlying psychological condition including various mood disorders and psychological trauma [3]. It is also known that 10% of patients who experience PNES suffer from a known seizure disorder [4]. Diagnosis of PNES and deciphering it from its non-psychogenic seizure counterpart can be challenging in the clinical setting. The diagnosis of psychogenic versus other seizure types can be made with the help of several clinical signs. These include gradual onset of seizure, vocalizations, side-to-side head movements, bilateral nonsynchronous movements, fluctuating course, and violent movements [8]. PNES can also present with unresponsiveness to various stimuli that can be similar to an absence seizure. Although absence seizures are more likely seen in childhood, they may initially present or recur in adulthood. Absence seizures typically present with behavioral arrest, blank facial expression, and impaired consciousness. If they last longer than 20 seconds, they are considered atypical absence seizures, which can have abnormal muscle tone and movements in addition to impaired consciousness. PNES typically last longer in duration compared to epileptic and absence seizures. In addition, absence and atypical absence seizures have abnormal EEG tracings, which, in an appropriate setting, can help differentiate from a psychogenic seizure [9]. The duration of the impaired consciousness this patient experienced was difficult to calculate given the rapid induction of anesthesia prior to the emergency cesarean section. Psychogenic etiology is favored when a patient presents with somatic activity resembling a seizure but does not respond to anti-epileptic drugs and there is no evidence of abnormal EEG tracings [10]. Signs to look for when differentiating epilepsy and psychogenic non-epileptic seizures are included in Table 1.

**Table 1:** Signs that may indicate a diagnosis when comparing epilepsy and psychogenic non-epileptic seizures (+)=more commonly seen; (-)=less commonly seen

Sign	Epilepsy	PNES
Emotional/Stress Trigger [10]	-	+
Psychiatric Comorbidities [10]	-	+
Epileptiform Activity (EEG) [10]	+	-
Tongue Biting [11]	+	-
Rapid Postictal Recovery [11]	-	+
Eyes Closed [11]	-	+
Duration [11]	<5 minutes	>5 minutes

Upon further questioning the patient in this case, a prior history of a similar incident of unresponsiveness of unknown duration occurring while at home during their first pregnancy was revealed. If the patient had been more forthcoming about her history during pregnancy, the disclosed information could have aided in the early diagnosis of PNES and potentially prevented the increased blood loss and prolonged intubation.

The day after the delivery, the patient also reported having severe pain during labor contractions, diffuse body paresthesia, and lightheadedness prior to going unresponsive. In the setting of these symptoms and an unremarkable postoperative workup with no prior epileptic history, there was a high suspicion of PNES in this patient. Additionally, on postoperative day two, she experienced an episode of lightheadedness and became tremulous, anxious, and confused. With concerns of another related episode, an extensive workup was performed, which again was unremarkable. Using this information and the patient’s prior history, the diagnosis of PNES was given. As a diagnosis of exclusion, PNES can incorporate a variety of patient presentations. To further aid in this diagnosis, the patient’s episodes of seizure-like activity displayed no abnormal tracings on an EEG that would be suggestive of a non-psychogenic seizure. 

There are limited reports describing pregnancy-related PNES diagnosis and treatment. Those that are available describe a history of psychiatric disorders, prior pregnancy loss, and significant stress during the pregnancy can be risk factors for provoking PNES [5]. Looking further into the current literature, the treatment for PNES focuses on managing any underlying psychiatric disorders and includes cognitive behavioral therapy, mindfulness strategies, and potentially pharmacotherapy if underlying mood disorders are present [10]. Recurrent PNES during pregnancy is frequently treated with benzodiazepines, anti-epileptic drugs, and even barbiturates despite a formal diagnosis of psychogenic seizures [11]. This greatly increases the patient's risk of pregnancy-related complications and fetal malformations. While little has been reported regarding psychogenic seizures during pregnancy, an increased understanding of the disorder and the patient’s history is imperative to differentiating it from other types of seizures. Early detection and proper treatment can prevent intrapartum episodes that may prompt further pharmacological or surgical intervention. PNES should be included in the differential when evaluating a patient with a seizure to allow a more systematic approach to the patient's care [9]. While these patients should be evaluated for epilepsy, eclampsia, and absence seizures, PNES may mimic the clinical presentation and should be considered before treatment.

## Conclusions

Psychogenic non-epileptic seizure (PNES) is not well-recognized in laboring women. These seizures are somatic manifestations, often associated with conversion disorders, and are more common in patients with a history of psychiatric conditions. PNES is characterized by convulsions and unresponsiveness to stimuli. PNES has subtle motor and sensory symptoms that resemble other neurological conditions, so it is considered a diagnosis of exclusion. It can resemble absence seizures, which are characterized by a lack of response or abrupt impairment of consciousness. This report presented the case of a 26-year-old laboring female who experienced multiple psychogenic seizures. The purpose of this case report is to raise awareness about a condition that is not commonly known, which may mimic epilepsy, absence seizures, and eclampsia. It is important to include this condition in the differential diagnosis to better treat the patient’s symptoms and avoid unnecessary procedures that may increase maternal morbidities.
